# Hydroxyl Functionalization Effects on Carbene–Graphene for Enhanced Ammonia Gas Sensing

**DOI:** 10.3390/molecules30244726

**Published:** 2025-12-10

**Authors:** Athar A. Hassanian, Kamal A. Soliman, Tawfiq Hasanin, Abdesslem Jedidi, Adnene Dhouib

**Affiliations:** 1 Chemistry Department, College of Science, Imam Abdulrahman Bin Faisal University, Dammam 31113, Saudi Arabia; 2 Department of Chemistry, Faculty of Science, Benha University, Benha P.O. Box 13518, Egypt; 3 Department of Information Systems, King Abdulaziz University, P.O. Box 80203, Jeddah 21589, Saudi Arabia; 4 Department of Chemistry, Faculty of Science, King Abdulaziz University, P.O. Box 80203, Jeddah 21589, Saudi Arabia; 5 FEMTO-ST Institute, CNRS, Université de technologie de Belfort Montbéliard, 90010 Belfort, France

**Keywords:** hydroxylation, DFT, functionalized graphene, NH_3_ adsorption

## Abstract

DFT study of graphene functionalized via carbene was performed to identify the preferred –OH adsorption sites and to assess how hydroxylation affects adsorption of NH_3_ gas. The carbene attaches to the graphene basal plane through a [2+1] cycloaddition, producing a local cyclopropane-like motif with a C–C bond. This modification introduces localized mid-gap states and asymmetric charge redistribution that create chemically active anchoring sites for –OH groups. We systematically scanned possible –OH adsorption sites and identified site-dependent binding energies. NH_3_ preferentially anchors at the carbene center and is further stabilized by multidentate hydrogen bonding with neighboring –OH groups. Calculated NH_3_ adsorption energies range from moderate values (single –OH and some two –OH symmetric sites, E_ads_ ≈ −0.64 to −0.75 eV) to strong interaction for selected through-plane two –OH pairs (E_ads_ ≈ −1.78 to −1.83 eV), where synergistic hydrogen bonding amplifies the NH_3_ interaction. Charge density difference and Bader analyses indicate polarization-dominated binding with minimal net charge transfer, consistent with hydrogen bonding rather than covalent bond formation. Desorption time estimation shows that moderate binding motifs provide rapid recovery at room temperature. We conclude that targeted placement of paired –OH groups on carbene-functionalized graphene offers a tunable route to balance sensitivity and reusability for NH_3_ sensing.

## 1. Introduction

Ammonia plays a vital role in the global nitrogen cycle. Atmospheric dinitrogen (N_2_) is biologically converted into reactive nitrogen through microbial nitrogen fixation, transformed through soil and water processes, and returned to the atmosphere as N_2_ or nitrogenous gases. This natural progression from N_2_ to NH_3_ and subsequently to oxidized nitrogen species supports ecosystem productivity and regulates the movement of reactive nitrogen within the environment. However, human activities have significantly elevated reactive nitrogen levels, increasing ammonia emissions and intensifying their effects on air quality, water eutrophication, and public health. Viewing NH_3_ within both its biogeochemical origins and its large scale industrial production highlights the importance of developing highly sensitive and selective ammonia sensors for environmental, agricultural, and industrial applications [1].

Ammonia (NH_3_), a pungent gas, widely used chemical in agriculture, fertilizer production, refrigeration, and many chemical industries, it is naturally found in soil and air [2] and poses serious health and environmental risks at elevated concentrations. Inhalation of NH_3_ above ~25 ppm can irritate the eyes, skin, and respiratory tract, and long-term exposure may lead to more severe health problems. Monitoring NH_3_ in ambient air, industrial exhaust, and even biological exhalations is therefore critically important. The release of high concentrations of ammonia also contributes to atmospheric pollution and deteriorates air quality, so there is an urgent need for gas sensors with high sensitivity and selectivity toward NH_3_. A wide range of sensing materials has been explored, including metal oxides [3,4,5,6], resistive sensors [7,8], and various two-dimensional (2D) nanomaterials [9,10,11]. Among these, 2D materials have attracted particular interest because of their very large specific surface area [12,13], excellent electrical conductivity [14,15], and high thermal conductivity [16,17], all of which are favorable for fast, sensitive gas detection. The isolation of single-layer graphene from graphite by Geim and Novoselov in 2004 marked the beginning of a new era for 2D materials research [18], and since then researchers have investigated a broad family of 2D systems to further expand sensing capabilities and application prospects [19,20,21,22,23,24,25].

Solid-state gas sensors based on changes in electrical conductivity when gas molecules adsorb onto a surface are promising for compact, low-power, real-time detection. Among sensing platforms, graphene- and graphene-derived materials are particularly attractive due to their ultra-thin structure, high carrier mobility, large surface area, and strong sensitivity to changes in surface adsorption [26]. However, pristine graphene (pure sp^2^ carbon sheet) interacts only weakly with small gas molecules like NH_3_, yielding low adsorption energies, limited charge transfer, and thus modest sensing responses [27]. To overcome that limitation, a variety of functionalization strategies have been explored: introducing defects, doping with heteroatoms, anchoring metal or metal-oxide nanoparticles, or grafting organic functional groups onto graphene or graphene oxide [28]. Among those, covalent functionalization (i.e., forming chemical bonds to the carbon lattice) can introduce local binding sites and perturb the electronic structure, thereby enhancing adsorption energy and sensitivity [29].

One particularly interesting functionalization route is via carbenes (divalent carbon species) or their precursors. Graphene functionalized by activated carbenes can produce covalent adducts on the graphene basal plane via [2+1] cycloaddition, thereby creating localized sites with chemical reactivity [30]. In a DFT study, Aldulaijan et al. used diazomethanes functionalized by –OH, –OMe, and –OEt, which after carbene attachment to graphene gave derivatives that showed significant adsorption affinity toward toxic gases (including NH_3_) via hydrogen bonds [31]. The same work emphasizes that the –OH functional group (hydroxyl) can act as a hydrogen bond donor/acceptor, thus enabling stronger interactions with NH_3_ [31]. Other computational studies have also explored substituted carbene grafting to graphene. For example, Baachaoui et al. simulated thermodynamics of graphene functionalization via substituted carbenes and assessed the resulting graphene adducts for gas adsorption, concluding that the nature of substituents plays a key role in modulating adsorption characteristics [32]. The controlled grafting of carbenes thus offers a tunable palette of functional sites on graphene surfaces. Once –OH groups are incorporated, there is potential for hydrogen bonding with NH_3_. Hydroxyl groups on graphene or graphene oxide are known to increase sensitivity and selectivity for NH_3_ detection [33]. For instance, plasma treatments of graphene can introduce oxygen-containing functional groups (hydroxyl, epoxide, carbonyl), and the modified graphene shows enhanced NH_3_ sensing performance compared to pristine graphene [34]. In particular, Iwakami et al. demonstrated that oxygen plasma treatment (introducing hydroxyl groups) enhances NH_3_ adsorption and sensor response [35]. Furthermore, the presence of –OH groups may affect not only binding strength but also kinetics and reversibility of adsorption/desorption, sensor recovery, and even selectivity [36]. Indeed, some works have shown that tuning the density (or selective removal) of hydroxyl groups influences gas sensitivity [37].

In the current study, we explored the sensing behavior of an –OH group functionalized carbene–graphene surface toward ammonia (NH_3_) gas using first-principles calculations. The aim of this work is to design and model a graphene–carbene-OH-functionalized surface and to evaluate its potential as a highly sensitive and selective NH_3_ gas sensor. To achieve this, we computed the adsorption energies, charge transfer, density of states (DOS), and optimized binding geometries of NH_3_ molecules on the proposed surface.

## 2. Results and Discussion

### 2.1. Graphene Functionalization with Carbene and Hydroxyl Groups

**Figure 1:**
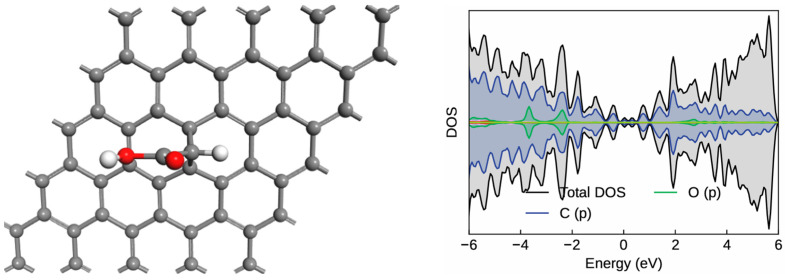
The optimized geometry and density of states of carbene–graphene surface. The red, white, and gray balls indicate oxygen, hydrogen, and carbon atoms, respectively.

The functionalization of graphene with carbene and hydroxyl groups was investigated systematically to understand the structural stability and electronic behavior of the resulting nanostructures. The initial step involved studying the interaction of the carbene moiety with the graphene surface through a [2+1]-cycloaddition reaction, as shown in Figure 1. In this configuration, the carbene functional group reacts with the π-conjugated carbon network of graphene to form a cyclopropane-type structure, confirming successful attachment of the carbene fragment. The optimized geometry of this configuration reveals C–C bond lengths ranging from 1.53 Å to 1.56 Å, which are characteristic of sp^3^-sp^3^ carbon–carbon single bonds. These bond distances are notably longer than the C=C bonds in pristine graphene (1.42 Å), indicating the partial transformation from sp^2^ to sp^3^ hybridization at the carbene attachment site. The calculated adsorption energy of −1.67 eV further confirms that this modification is energetically favorable and in agreement with previously reported values for carbene–graphene cycloadditions [27,28].

The graphene–carbene nanostructure consists of 52 carbon atoms, 2 hydrogen atoms, and 2 oxygen atoms, forming a nearly planar configuration with localized distortion near the carbene site. The density of states (DOS) profile, also shown in Figure 1 and Figure S1, reflects the metallic or semi-metallic nature of the system. Pristine graphene displays a finite band gap, whereas carbene-functionalized graphene becomes metallic as new defect states emerge near the Fermi level (Figure S1). The electronic states near the Fermi level are primarily derived from the p-orbitals of carbon atoms, which maintain the conductive π-network typical of graphene. The introduction of the carbene moiety slightly perturbs this delocalized π-system, giving rise to localized mid-gap states and asymmetric charge redistribution around the carbene site. This perturbation creates electronic inhomogeneity, enhancing the reactivity and adsorption potential of nearby carbon atoms. These localized regions of charge density serve as ideal anchoring sites for further surface functionalization, such as hydroxyl (–OH) attachment.

**Figure 2:**
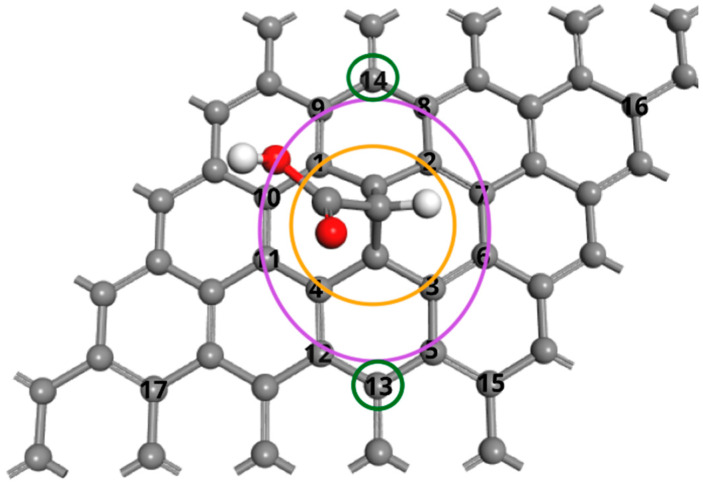
The adsorption positions (1–17) of the –OH group for the carbene–graphene surface. Orange circle for position first neighbor to carbene, purple circle is second neighbor, and green is third neighbor.

Building upon this structure, the adsorption of a single –OH group on various carbon sites of the graphene–carbene surface was systematically investigated. Figure 2 displays the seventeen possible adsorption sites of the –OH group, both above and below the graphene plane. For clarity, the positions were divided into three coordination zones based on their spatial relationship to the carbene center. The first coordination (C1–C4) is closest to the carbene, second coordination (C5–C12) is intermediate, and the third coordination (C13–C14) is farthest from the carbene (Figure 2). This partitioning reveals how local geometric strain and electronic perturbation produced by the carbene control adsorption energetics and geometry.

When the –OH group was placed above the graphene plane, the lowest (most negative) binding energies were found at the third coordination sites C13 and C14 (E_b_ ≈ −1.48 eV), showing that these distal sites are the most favored for single –OH binding (Table 1). The first coordination sites (C1–C4) are the next most favorable (E_b_ ≈ −1.24 to −1.31 eV), whereas most second coordination positions give substantially weaker binding (≈ −0.8 to −1.02 eV). The reduced lattice strain and fewer local constraints at the third coordination carbons make them energetically preferred, even though they are farther from the electronically perturbed carbene center and proximity to the carbene does not always increase stability; instead, a balance between electronic activation (favoring nearby sites) and local steric determines E_b_.

Table 1 also shows binding energies for below-plane placements; several below-plane values are more negative than their above-plane counterparts (the E_b_ of –OH group below C3 is −1.76 eV, but above C3 is −1.31 eV). This systematic strengthening for some sites when the –OH is placed below indicates that the three-dimensional orientation of the –OH relative to the carbene moiety can favor stronger H-bonding or improved overlap with the graphene π -cloud when the group is beneath the sheet. In particular, below-plane geometries often place the hydroxyl hydrogen in a geometry that better complements the lone-pair/partial negative site on the carbene oxygen, yielding stronger intramolecular hydrogen bonding and thus more negative adsorption energies.

The most stable single-OH adsorptions above the plane occur at C13 and C14 (E_b_ = −1.48 eV); the most stable below-plane example is C3 below (E_b_ = −1.76 eV). Structural parameters extracted from the relaxed geometries (Table 2) reveal the microscopic origin of these stabilities. The covalent C–O distance for the adsorbed hydroxyl is ~1.48 Å (d6), as seen in Table 2, while the O–H bond is ~0.98 Å (d5) in all most stable configurations. The presence of a hydrogen-bond-like contact between the hydroxyl hydrogen and the carbene oxygen contributes significantly to stabilization for the third-coordination sites (C13 and C14). This intramolecular interaction is geometrically feasible at distal sites because the graphene sheet can relax locally without excessive strain. The C–O–H angle at the stable third-coordination sites is ≈108.6°, consistent with sp^3^-like geometry around the oxygen and with minimal angular strain. The angles α, β, and $\gamma_{1}$ reported in Table 2 change only mildly between C13 above and C14 above, indicating only small local distortions despite the formation of a new covalent bond. Also, Table 2 lists vertical offsets that quantify how far the functionalized carbon and substituent sit relative to the graphene plane. For above-plane binding, the local atom carrying the –OH is displaced outward by ≈0.56–0.66 Å, whereas for below-plane adsorption, the corresponding displacement can be negative (h1 ≈ −0.31 Å), reflecting the group’s orientation beneath the sheet. These values show that local puckering accommodates the adsorbate and is larger for above-plane bonding than for some below-plane geometries. This out-of-plane relief is an important component of the adsorption energy balance because it mediates strain in the sp^2^ lattice.

**Figure 3:**
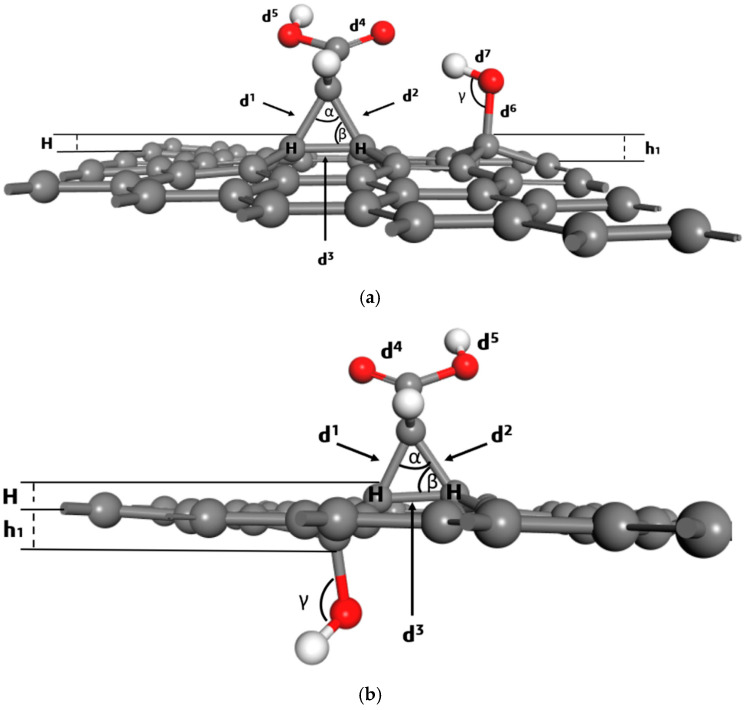
Symbols of the geometric parameters after OH group adsorption in (**a**) position 14 above and (**b**) position 3 below the carbene–graphene surface.

Top and side views of the representative stable geometries (Figures S2 and S3) for above- and below-plane confirm the numerical interpretations; at C13/C14 above the –OH adopts a nearly upright geometry, forming a favorable H-bond to the carbene oxygen (Figure S2e,f). At C3 below the –OH can approach the carbene oxygen from beneath, enabling even shorter H-bond contacts and a slightly more negative adsorption energy (Figure S3c). The geometric symbols labeled in Figure 3 help to visualize the measured distances and angles summarized in Table 2. These images corroborate that combination of optimal bond determines why certain distal sites outperform closer ones energetically. This site sensitivity implies that selective functionalization of graphene–carbene could be guided for designing sensors or catalyst supports where defined placement of polar groups controls the adsorption of probe molecules (NH_3_) or modifies local electronic properties. The local DOS perturbations introduced by the carbene and by the adsorbed –OH will also influence charge transfer with adsorbates and thus the surface’s chemical selectivity.

**Figure 4:**
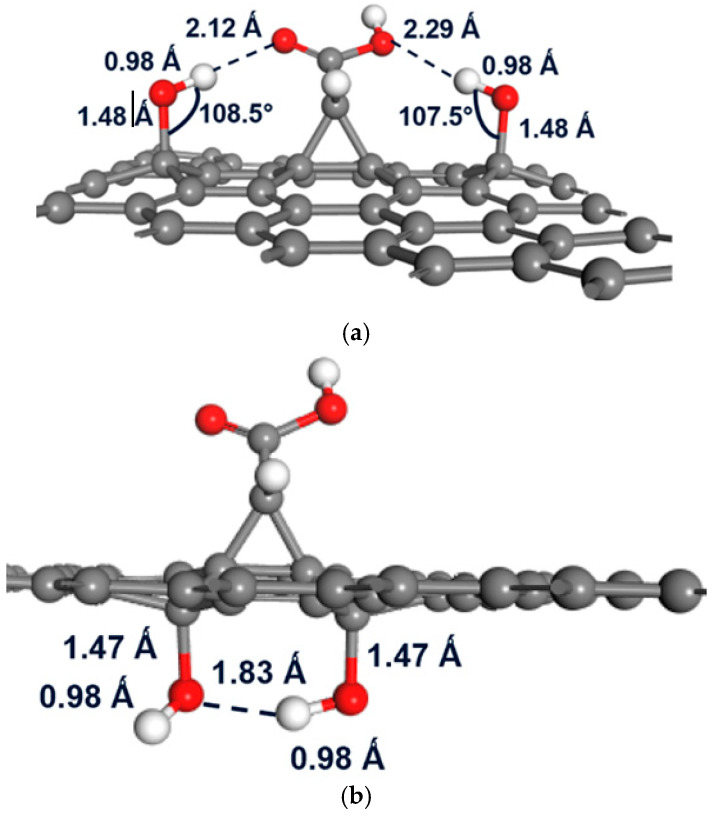
Highest binding energy for a two –OH group (**a**) on top (position C13–C14) and (**b**) below (position C2–C3) the carbene–graphene surface.

When a second hydroxyl is introduced on the graphene–carbene surface, the binding landscape changes markedly compared with the single –OH case. Tables S1–S3 show three different two –OH scenarios: first, both –OH placed above the sheet (C13 and C14), as presented in Table S1. The second case is one –OH fixed above (C13 or C14) and a second –OH explored below over all other sites, as shown in Table S2. The third case is one –OH fixed below (C3) with the second –OH scanned below over all sites, as seen in Table S3. Stronger binding energies are obtained for many two –OH combinations versus the single –OH results. For example, placing –OH at both C13 and C14 above yields E_b_ ≈ −1.97 eV (Table S1), which is significantly more stabilizing than a single –OH at C13 or C14. When one –OH is fixed at an above site (C13 or C14) and the second –OH is placed at below sites, the E_b_ values often reach ~−2.09 to −2.14 eV for the most favorable combinations (Table S2), demonstrating cooperative stabilization between the two substituents. In the case where the first –OH is fixed below (C3), scanning the second –OH below (Table S3) gives an extreme stabilization at C2 (E_b_ ≈ −2.50 eV), marking the strongest binding reported across these scans. The corresponding optimized geometries are depicted in Figure 4 and Figure S4.

Figure 4 highlights the dominant –OH group above-plane pairing at C13 and C14 (Figure S5 labels the geometric parameters that define this symmetric configuration), and emphasizes the exceptionally stable C2 with C3 below configuration, where the through-plane complementarity between the two –OH groups produces the strongest binding energy of −2.50 eV.

Figure S4 illustrates how fixing one –OH above the surface (C13 or C14) biases the electronic environment toward nearby carbons, such as C1–C4, enabling cooperative stabilization through electronic and hydrogen-bond interactions.

The markedly enhanced stability of two –OH functionalization on graphene–carbene arises from three cooperative effects, short O···H contacts (≈1.8–2.2 Å) and C–O–H angles (~108°) that produce inter-/intra-molecular hydrogen bond networks, charge redistribution that polarizes neighboring carbons and lowers the barrier for a second binding event, and strain sharing between adjacent sp^3^ centers that reduces the lattice distortion penalty, together explaining why paired motifs are far more stable than isolated OH (single –OH/carbene reference ≈ −1.67 eV). This mechanistic picture is reflected in the structure energy correlations; the symmetric above-plane C13/C14 pair (Table 3 and Table S1; Figure 4a and Figure S4) exhibits C–O distances of 1.52–1.53 Å, O–H ≈ 0.98 Å and nearly equal out-of-plane displacements (h_1_ ≈ h_2_ ≈ 0.56 Å), accounting for the additional ≈ 0.5 eV stabilization (E_b_ ≈ −1.97 eV); fixing one OH above (C13 or C14) and placing the second in nearby below-plane sites (Table S2 and Figure S4) yields directional cooperativity with E_b_ down to ≈−2.09 to −2.21 eV as dipole alignment and local bending favor specific partners; and the extreme case of fixed C3 below with a second OH placed below C2, as seen in Table S3 and Figure 4b, achieves E_b_ ≈ −2.50 eV via a through-plane O–H⋯O “handshake” that simultaneously optimizes dipole coupling and minimizes strain (h_1_ < 0, h_2_ > 0). These computed magnitudes and motifs are consistent with prior DFT reported by Baachaoui et al. [28] for carbene cycloaddition energetics. Tran et al. [38] demonstrated that neighboring –OH groups on graphene stabilize each other via hydrogen-bonded networks with O⋯H distances of 1.8–2.1 Å, mirroring the short contacts calculated in this study. Similarly, Mouhat et al. [39] reported multi-hydroxyl arrangements in graphene oxide sheets that yield strong cooperative stabilization, comparable to the 0.5–1.0 eV gain per group seen for the C13 and C14 pair. Prasert et al. [40] and Boukhvalov [41] further highlighted that clustering of oxygen functionalities reduces local strain and minimizes puckering energy, mechanisms that explain the nearly equal out-of-plane displacements (h_1_ ≈ h_2_ ≈ 0.56 Å) found in Table 3. The exceptionally strong binding of the C3 below and C2 below configuration (Table S3 and Figure 4b) is analogous to the cooperative adsorption patterns observed in DFT simulations of hydroxylated graphene oxide layers, where dipole alignment across the sheet lowers total energy. Overall, these comparisons confirm that the enhanced stabilities computed in Table 3 and Tables S1–S3 arise from the same synergistic effects, hydrogen bond networking, charge polarization, and strain redistribution that have been consistently identified in theoretical and experimental studies of oxygen-functionalized graphene systems, implying that the strategic placement of paired OH groups can be used to tune local DOS, enhance charge transfer, and optimize performance in applications such as gas sensing.

### 2.2. NH_3_ Gas Sensing on the OH-Modified Graphene–Carbene Surface

**Figure 5:**
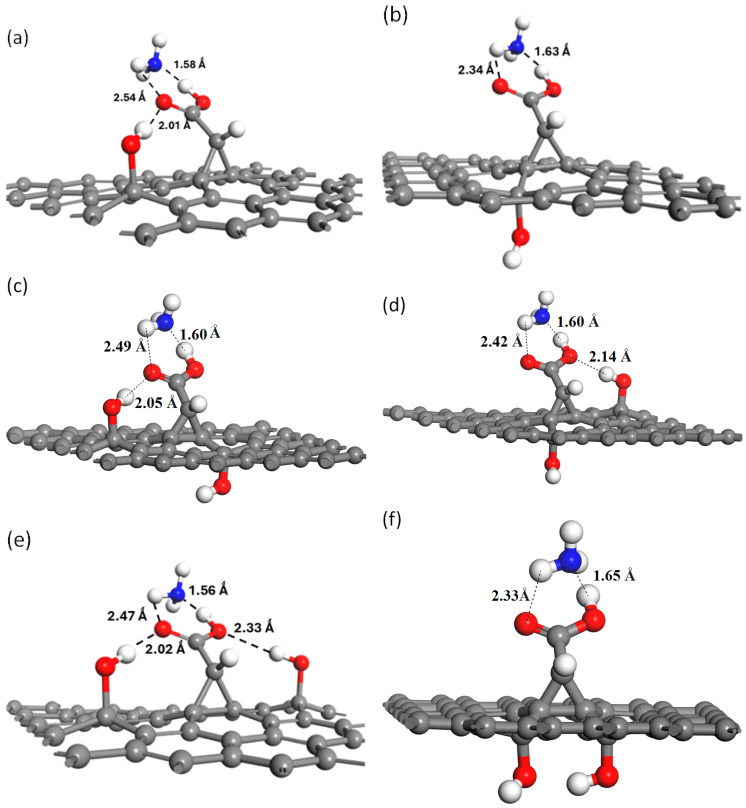
NH_3_ adsorption on the most stable sites for –OH groups on the modified carbene–graphene surface with 1OH (**a**) above C13, (**b**) below C3, and 2OH (**c**) above C13 and below C2, (**d**) above C14 and below C3, (**e**) above both C13 and C14, (**f**) below both C3 and C2.

The adsorption and sensing behavior of NH_3_ on the –OH-functionalized graphene–carbene surface was systematically investigated, as presented in Table 4. A full structural optimization was performed as seen in Figure 5, for each configuration to identify the most energetically favorable arrangement of NH_3_ molecules on both single –OH- and two –OH-functionalized graphene–carbene surfaces. NH_3_ preferentially adsorbs on the carbene center of the OH-functionalized graphene–carbene surface because of the unique combination of electronic, structural, and chemical properties introduced by both the carbene moiety and the adjacent hydroxyl groups. The carbene site in graphene–carbene possesses a highly localized and partially empty p orbital, making it electron-deficient and chemically active. This orbital can interact directly with the lone pair of electrons on the nitrogen atom of NH_3_, forming a weak coordinate bond. Such an interaction is analogous to Lewis acid–base behavior, where the carbene carbon acts as a Lewis acid and the NH_3_ molecule serves as a Lewis base. This explains why the carbene site provides a natural anchoring point for NH_3_, resulting in stronger adsorption than on pristine graphene, which has delocalized π-electrons and lacks localized reactive centers. In this arrangement the nitrogen lone pair is oriented toward the hydroxyl group of the carbene-functionalized graphene moiety, producing a clear N⋯H–O interaction, while simultaneously one of the N–H hydrogens of NH_3_ forms an H⋯O contact with the oxygen atom of the same carboxyl group. At the same time, the oxygen atom of the same carboxyl group of the carbene-functionalized graphene moiety engages with the neighboring surface –OH group (from the functionalized graphene–carbene), forming an O–H⋯O interaction that links the carbene moiety and the surface OH into a cooperative scaffold. These three contacts create a multidentate binding pocket that both locks NH_3_ in a favorable orientation and distributes the interaction energy across several directional bonds. Table 4 shows that NH_3_ adsorption on the OH-modified graphene–carbene surface is highly site-dependent. A single OH (above C13 or below C3) binds NH_3_ moderately (E_ads_ ≈ −0.70 to −0.64 eV), whereas certain two-OH arrangements produce dramatically stronger adsorption; the pair above C13 and below C2 and the pair above C14 and below C3 give E_ads_ ≈ −1.78 and −1.83 eV, respectively (Table 4). By contrast, the symmetric pair above C13 and C14 and the pair below C3 and C2 show weaker NH_3_ adsorption (E_ads_ ≈ −0.75 and −0.62 eV). These trends indicate that NH_3_ interacts most strongly with two-OH geometries that provide a complementary, through-space interaction geometry. The distance data (Table 4) and the top/side views in Figure 5 show that NH_3_ is held by true hydrogen bonds rather than weak physisorption, the N⋯H contacts lie very short (≈1.56–1.65 Å), while the H⋯O contacts to carbonyl/hydroxyl oxygens fall in the ~2.0–2.5 Å range, all consistent with moderate–strong H-bonding.

**Figure 6:**
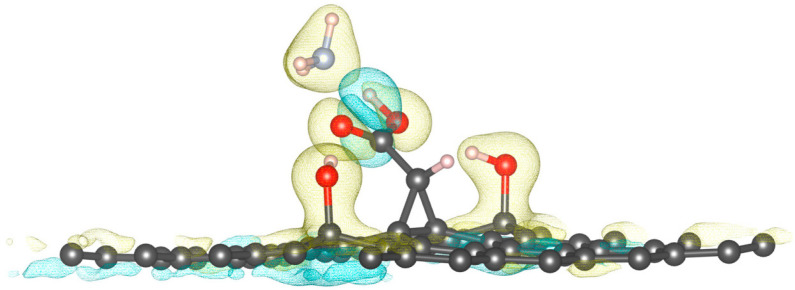
Charge density difference of NH_3_ on two –OH groups, above C13 and C14, on modified carbene–graphene surface. The yellow regions represent electron accumulation, whereas the blue regions indicate electron depletion.

The charge density difference (CDD) analysis further supports these findings for the two–OH-functionalized graphene–carbene system, which exhibits an adsorption energy of approximately −0.75 eV and a relatively short desorption time, confirming a reversible physisorption-type interaction suitable for sensing applications. The CDD plot (Figure 6), where yellow areas represent electron accumulation and cyan areas represent electron depletion, reveals localized regions of electron accumulation around the nitrogen atom of NH_3_ and depletion near the oxygen atoms of the hydroxyl and carboxyl groups, indicating polarization-driven charge redistribution rather than covalent bonding. Correspondingly, the Bader charge values listed in Table 4 (Q ≈ −2.88 to −2.92 e) remain nearly constant across adsorption motifs, showing that no significant electron transfer occurs between NH_3_ and the substrate. This suggests that the adsorption mechanism is dominated by hydrogen bonding and electrostatic polarization, consistent with the moderate adsorption energy and fast desorption observed. In essence, NH_3_ binds through localized, multidentate hydrogen bonds (N⋯H–O and H⋯O=C) while maintaining electronic reversibility, an essential feature for a sensitive yet easily recoverable gas sensor.

**Figure 7:**
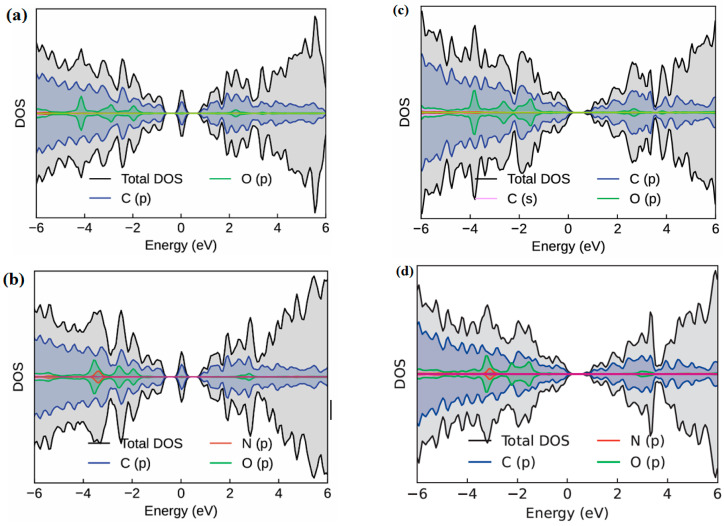
DOS of –OH groups modified graphene–carbene surface before and after NH_3_ adsorption: (**a**) –OH group above C13 before NH_3_ adsorption, (**b**) –OH group above C13 after NH_3_ adsorption, (**c**) –OH group above C13 and C14 before NH_3_ adsorption, (**d**) –OH group above C13 and C14 after NH_3_ adsorption.

The DOS analysis of OH-modified graphene–carbene (Figure 7) shows how NH_3_ adsorption alters the local electronic structure in ways that directly connect to sensor response and to expected desorption behavior; for the single OH (above C13) case (Figure 7a,b) NH_3_ adsorption produces small but clear spectral changes, peaks associated with O-p and nearby C-p states shift slightly, and weak, localized molecular states from the NH_3_ lone pair appear just below the Fermi level. These changes are consistent with modest donor–acceptor coupling (NH_3_ → surface), a small upward shift in electronic density near E_F_, and only minor broadening of the bands, which together explain the moderate adsorption energy (≈−0.64 to −0.70 eV) and the relatively fast desorption/recovery expected at room temperature. In contrast, the two OH cases show a split behavior; the symmetric above-plane pair (C13 and C14) (Figure 7c,d) induces somewhat larger DOS perturbations than the single OH, and new features near E_F_ become sharper and slightly more intense, yet remain moderate in magnitude (E_ads_ ≈ −0.75 eV), implying enhanced sensitivity but still facile desorption. The through-plane, multidentate arrangements produce the largest DOS modifications and pronounced new peaks, and greater hybridization between NH_3_-derived states and O-p/C-p orbitals appear near the Fermi level, indicating stronger orbital overlap and larger polarization. Charge density difference maps (Figure 6) corroborate this picture by showing localized electron accumulation on the NH_3_ lone pair and complementary depletion on the donor oxygens, which explains the DOS signatures. Practically, single-OH and symmetric two-OH sites provide measurable but reversible electronic signals, whereas through-plane two-OH pockets give much larger DOS perturbations.

### 2.3. Desorption Time (τ)

The desorption time (τ) represents the duration required for NH_3_ molecules to detach from the surface and for the sensor material to return to its original state, a key indicator of reusability and sensor recovery speed. Desorption time was calculated using the Arrhenius-type relationship as follows [42]:

(1) $$\tau =\;\nu^{-1}{exp}^{\left(\frac{{-E}_{ads}}{k_{B}T}\right)}$$

where E_ads_ is the adsorption energy, ν is the attempt frequency ($10^{12}\;s)$, $k_{B}$ is the Boltzmann constant (8.62 $\times \;10^{-5}\;eV/K$), and T is the Temperature (300 K). Based on this expression, the computed desorption times for NH_3_ on different OH-functionalized graphene–carbene configurations are summarized in Table 4. The results demonstrate that single OH and some two-OH configurations (below-plane pairs) exhibit short desorption times, ensuring high reversibility and reusability as NH_3_ gas sensors. Conversely, configurations with through-plane OH pairs (above C13, below C2 and above C14, below C3) exhibit excessively long desorption times (≈10^18^ s), indicating nearly irreversible adsorption at room temperature. According to Xiong et al. [42], *τ* decreases exponentially with temperature, so increasing the operating temperature would dramatically reduce *τ* to practical levels. Therefore, moderate binding motifs such as single OH and the two-OH configurations (above C13, above C14) pairs provide the best compromise between sensitivity and reusability for NH_3_ gas sensing on graphene–carbene surfaces.

### 2.4. Comparison with Previous Studies

Several DFT studies, as seen in Table 5, show that pristine graphene binds NH_3_ only very weakly (Eads ≈ 14.7–30.8 meV), explaining its low intrinsic response. The interaction of NH_3_ with pyridinic nitrogen-doped graphene reported that the introduction of pyridine-type N defects enhances the chemical reactivity of the graphene surface. Density functional calculations show that NH_3_ adsorption becomes energetically favorable at these defect sites, with adsorption energies falling in the weak to moderate range, stronger than pristine graphene but still well below the chemisorption range. Functionalizations that introduce O-containing groups (hydroxyl, epoxide, COOH) or covalent anchoring sites (carbenes, carboxylated edges) markedly increase adsorption through hydrogen bonding and local electronic perturbation; reported behavior ranges from moderate (Eads ≈ 0.2–1.0 eV) to strong (Eads ≥ 1.0 eV). The present work provides the following results: Eads ≈ −0.64 to −1.83 eV (see Table 4). The single-OH motif remains in the moderate adsorption regime and thus maintains practical regeneration at near-room temperature, while the multidentate pocket shows strong adsorption and amplified sensing signals but requires elevated temperatures for desorption. This behavior is fully consistent with earlier DFT reports on covalently modified graphene, nitrogen-doped graphene, and carbene- or oxygen-functionalized graphene, all of which show that increased chemical anchoring leads to enhanced sensitivity at the cost of slower kinetics.

## 3. Materials and Methods

Electronic structure calculations for the –OH-functionalized carbene–graphene surface were performed using the Vienna Ab-initio Simulation Package (VASP, version vasp.5.4.4.pl2). The interaction between core and valence electrons was described by the projector-augmented wave (PAW) method [46], and spin-polarized density functional theory (DFT) calculations were carried out employing the revised Perdew–Burke–Ernzerhof (PBE) exchange–correlation functional within the generalized gradient approximation (GGA) framework [47]. The vdW interaction was performed using Grimme dispersion correction [48]. A plane-wave cutoff energy of 450 eV was used to accurately represent the valence electrons of the constituent atoms. A vacuum spacing of 20 Å was introduced along the z-direction to eliminate spurious interactions between periodic images. Structural optimization was performed until the forces on all atoms were less than 0.01 eV/Å, ensuring full relaxation of the geometry. The charge redistribution upon –OH functionalization and NH_3_ adsorption was quantified using Bader charge analysis, as implemented by the Henkelman Group [49]. The total and partial density of states (DOS) were generated using the Sumo package, while structural models and charge density difference plots were visualized through the VESTA software (ver. 3.90.5a) [50]. To investigate gas sensing behavior, NH_3_ adsorption was simulated at various high-symmetry sites of the –OH-functionalized carbene–graphene surface using the VESTA software [50]. A Monkhorst–Pack grid of 5  ×  5  ×  1 k-points was applied for structural optimization, and a denser 7  ×  7  ×  1 grid was employed for the electronic structure calculations. The binding energy (E_b_) of OH groups on the carbene–graphene surface, the adsorption energy (E_ads_) of NH_3_ molecules on the –OH-functionalized system, and charge density differences (CDD) were calculated using the following equations [51]:

(2) $$E_{b}=E_{surf+OH}-E_{surf}-E_{OH}$$

(3) $$E_{ads}=E_{surf+OH+NH3}-E_{surf+OH}-E_{NH3}$$

(4) $$∆\rho \left(r\right)=\rho_{surf+OH+NH3}\left(r\right)-\rho_{surf+OH}\left(r\right)-\rho_{NH3}\left(r\right)$$

where $E_{surf}$, $E_{surf+OH}$, $E_{OH}$, $E_{surf+OH+NH3}$, and $E_{NH3}$ are the total energy of the relaxed clean carbene–graphene surface, the total energy of the relaxed carbene–graphene surface with the adsorbed –OH group, the total energy of an isolated OH fragment, the total energy of the fully relaxed system with NH_3_ adsorbed on the –OH-functionalized surface, and the total energy of an isolated NH_3_ molecule, respectively. $\rho \left(r\right)\;$is the electron density of the corresponding relaxed system positioned in the same supercell and alignment.

## 4. Conclusions

Carbene functionalization of graphene generates highly reactive anchoring sites that profoundly alter both its geometry and electronic characteristics. Subsequent hydroxyl (–OH) adsorption on the carbene-modified surface displays strong site and spatial dependence. Below-plane –OH configurations on carbene–graphene are generally more stable than above-plane ones because they allow better hydrogen bonding and less lattice strain. When two –OH groups are added, they interact cooperatively through hydrogen bonds, further stabilizing the surface. The symmetric above pair (C13 and C14) increases stability by about 0.5 eV, while through-plane or below pairs can reach stronger binding of around −2.50 eV. NH_3_ adsorption on the –OH-functionalized surface is configuration-dependent, forming hydrogen-bond networks with nearby COOH groups. The adsorption energy varies from moderate (−0.64 to −0.75 eV) to strong (−1.78 to −1.83 eV), depending on site geometry. Charge analysis shows that hydrogen bonding and polarization dominate, with minimal charge transfer, confirming reversible physisorption. Desorption time calculations reveal that weak to moderate sites enable quick recovery, while strong through-plane sites bind almost permanently. Hence, configurations with computed longer desorption times will require explicit regeneration, such as multidentate carbene–OH pockets, which need hundreds of °C to thermally desorb NH_3_; therefore, practical implementations must include microheater or photo-assisted regeneration, or else use the weaker single–OH motif for continuous room temperature sensing to balance sensitivity and reversibility.

## Figures and Tables

**Table 1:** Binding energies (E_b_) of the 17 positions for OH groups above and below the carbene–graphene surface.

	Above OH Group	Below OH Group
	E_b_ (eV)	E_b_ (eV)
1	−1.24	−1.72
2	−1.31	−1.72
3	−1.31	−1.76
4	−1.24	−1.72
5	−0.88	−0.93
6	−1.00	−0.86
7	−1.00	−0.88
8	−0.83	−0.93
9	−0.89	−0.93
10	−0.81	−0.91
11	−0.81	−0.90
12	−1.01	−0.93
13	−1.48	−1.25
14	−1.48	−1.26
15	−0.80	−0.95
16	−1.02	−0.96
17	−0.90	−0.94

**Table 2:** The geometric parameters after modification on the carbene–graphene surface by –OH groups in the most stable case.

Geometric Parameters	–OH Above C13	–OH Above C14	–OH Below C3
E_b_ (eV)	−1.48	−1.48	−1.76
d1 (Å)	1.52	1.54	1.5
d2 (Å)	1.54	1.52	1.55
d3 (Å)	1.54	1.54	1.53
d4 (Å)	1.23	1.22	1.22
d5 (Å)	0.98	0.98	0.98
d6 (Å)	1.48	1.48	1.48
d7 (Å)	0.99	0.99	0.98
α (°)	60.2	60.3	60.2
β (°)	59.5	60.3	58.3
γ1 (°)	108.6	108.4	106.6
h1 (Å)	0.65	0.66	−0.31
H (Å)	0.60	0.56	0.50
	0.57	0.60	0.52

**Table 3:** The geometric parameters after modification of the carbene–graphene surface by a two –OH group in positions 13 and 14 above the surface.

Geometric Parameters	Value
E_b_ (eV)	−1.97
d1 (Å)	1.53
d2 (Å)	1.53
d3 (Å)	1.52
d4 (Å)	1.22
d5 (Å)	0.98
d6 (Å)	1.48
d7 (Å)	0.98
d8 (Å)	1.49
d9 (Å)	0.98
α (°)	58.8
β (°)	58.9
γ1 (°)	108.2
γ2 (°)	107.8
h1 (Å)	0.56
h2 (Å)	0.56
H (Å)	0.50
	0.52

**Table 4:** The adsorption energies (E_ads_), bond distances (d), and Bader charges of NH_3_ (Q) adsorbed on modified graphene–carbene surface: (a) –OH group above C13; (b) –OH group below C3; (c) two –OH groups above C13, below C2; (d) two OH groups above C14, below C3; (e) two OH groups above C13, above C14; and (f) two OH groups below C3, below C2.

System	Position	E_ads_ (eV)	d (Å)	Q	τ (s)
1OH	Above C13	−0.70	1.58 (N-H), 2.54 (H-O)	−2.89	5.7 $\times \;10^{-1}$
1OH	Below C3	−0.64	1.63 (N-H), 2.34 (H-O)	−2.91	6.5 $\times \;10^{-2}$
2OH	Above C13, below C2	−1.78	1.60 (N-H), 2.49 (H-O)	−2.89	7.82 $\times \;10^{17}$
2OH	Above C14, below C3	−1.83	1.60 (N-H), 2.42 (H-O)	−2.90	5.41 $\times {\;10}^{18}$
2OH	Above C13 and C14	−0.75	1.56 (N-H), 2.47 (H-O)	−2.88	3.94
2OH	Below C3 and C2	−0.62	1.65 (N-H), 2.33 (H-O)	2.92	2.58 $\times \;10^{-2}$

**Table 5:** Comparison adsorption of NH_3_ on 2D materials.

Modification/Functionalization Position	Reported NH_3_ Adsorption Range	Sensing Implication	Reference
Pristine graphene	Weak (physisorption)	Very low intrinsic sensitivity; rapid recovery	[43]
Graphene oxide (epoxide/hydroxyl)	Moderate to strong	Improved sensitivity; some GO sites show near-chemisorption	[44]
N-doped graphene	Weak to moderate	Better sensor response than pristine; still often reversible at room temperature	[45]
Carboxylated graphene nanoribbons	Moderate to strong	Improved sensitivity via engineered binding sites	[33]
Carbene functionalized graphene	Moderate to strong	Improved sensitivity via engineered binding sites	[28]
Graphene with functionalized carbenes	Moderate to strong	Potential selectivity	[27]

## Data Availability

The original contributions presented in this study are included in the article/Supplementary Material. Further inquiries can be directed to the corresponding authors.

## Supplementary Materials

The following supporting information can be downloaded at https://www.mdpi.com/article/10.3390/molecules30244726/s1. Figure S1. The optimized geometry and density of states of (a) pristine graphene and (b) carbene graphene surface. Figure S2. Top and side views for the most stable structures –OH group on above carbene graphene surface. Figure S3. Top and side views for the most stable structures –OH group on down carbene graphene surface. Figure S4. The highest binding energy positions for two OH group modified carbene graphene surface. Figure S5. Symbols of the geometric parameters after modification carbene graphene by two OH group in positions 13 and 14 above the surface. Table S1. Binding energy (Eb) for the 17 positions of two –OH group with (a) position 13 (b) position 14 modified carbene graphene surface. Table S2. Binding energy (Eb) for the 17 positions of two OH group above and below carbene graphene surface with (a) fixed position 13 above (b) fixed position 14 above. Table S3. Binding energy (Eb) for the 17 positions of two OH group modified carbene graphene surface with fixed position 3 below the surface.
