# Supplementary material for: Hydroxyl Functionalization Effects on Carbene–Graphene for Enhanced Ammonia Gas Sensing
Source: Molecules. 2025 Dec 10;30(24):4726. doi: 10.3390/molecules30244726 (PMC12736345; doi:10.3390/molecules30244726)
Supplement: Supplementary file 1 [file molecules-30-04726-s001.zip › molecules-4018524-supplementary.pdf]

Hydroxyl functionalization effects on carbene graphene for enhanced ammonia gas sensing

Athar Hassanian[a], Kamal A. Soliman[b], Tawfiq Hasanin[c], Abdesslem Jedidi[d,e*], Adnene Dhouib[a]

[a] Chemistry Department, College of Science, Imam Abdulrahman Bin Faisal University, Dammam 31113, Saudi Arabia

[b] Department of Chemistry, Faculty of Science, Benha University, P.O. Box 13518, Benha, Egypt

[c] Department of Information Systems, King Abdulaziz University, Jeddah, Saudi Arabia

[d] Department of Chemistry, Faculty of Science, King Abdulaziz University, P.O. Box 80203, Jeddah 21589, Saudi Arabia

[e] FEMTO-ST Institute, CNRS, Université de technologie de Belfort Montbéliard, Belfort, France

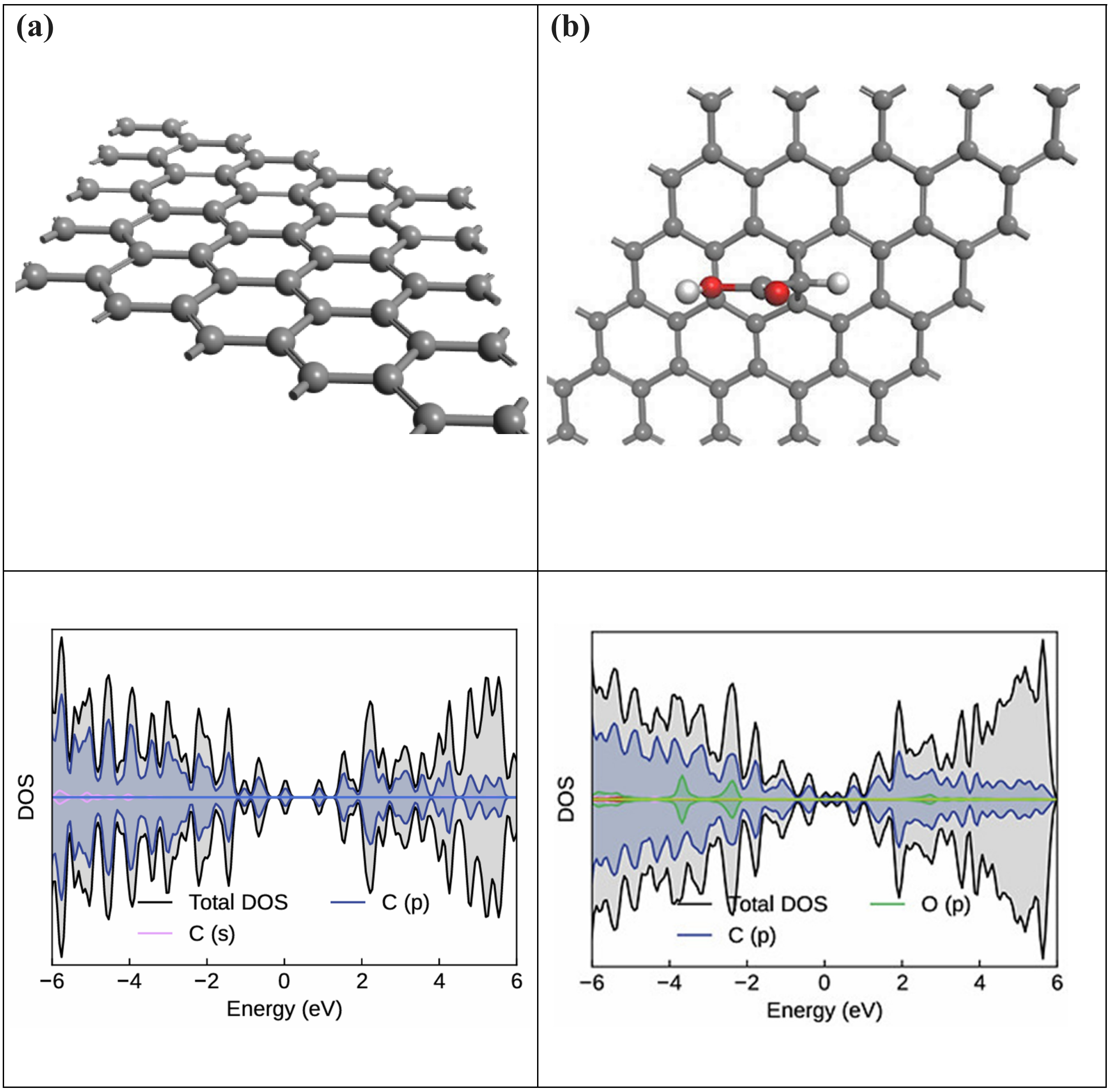

Figure S1. The optimized geometry and density of states of (a) pristine graphene and (b) carbene graphene surface. The red, white, and grey balls indicate oxygen, hydrogen, and carbon atoms, respectively.

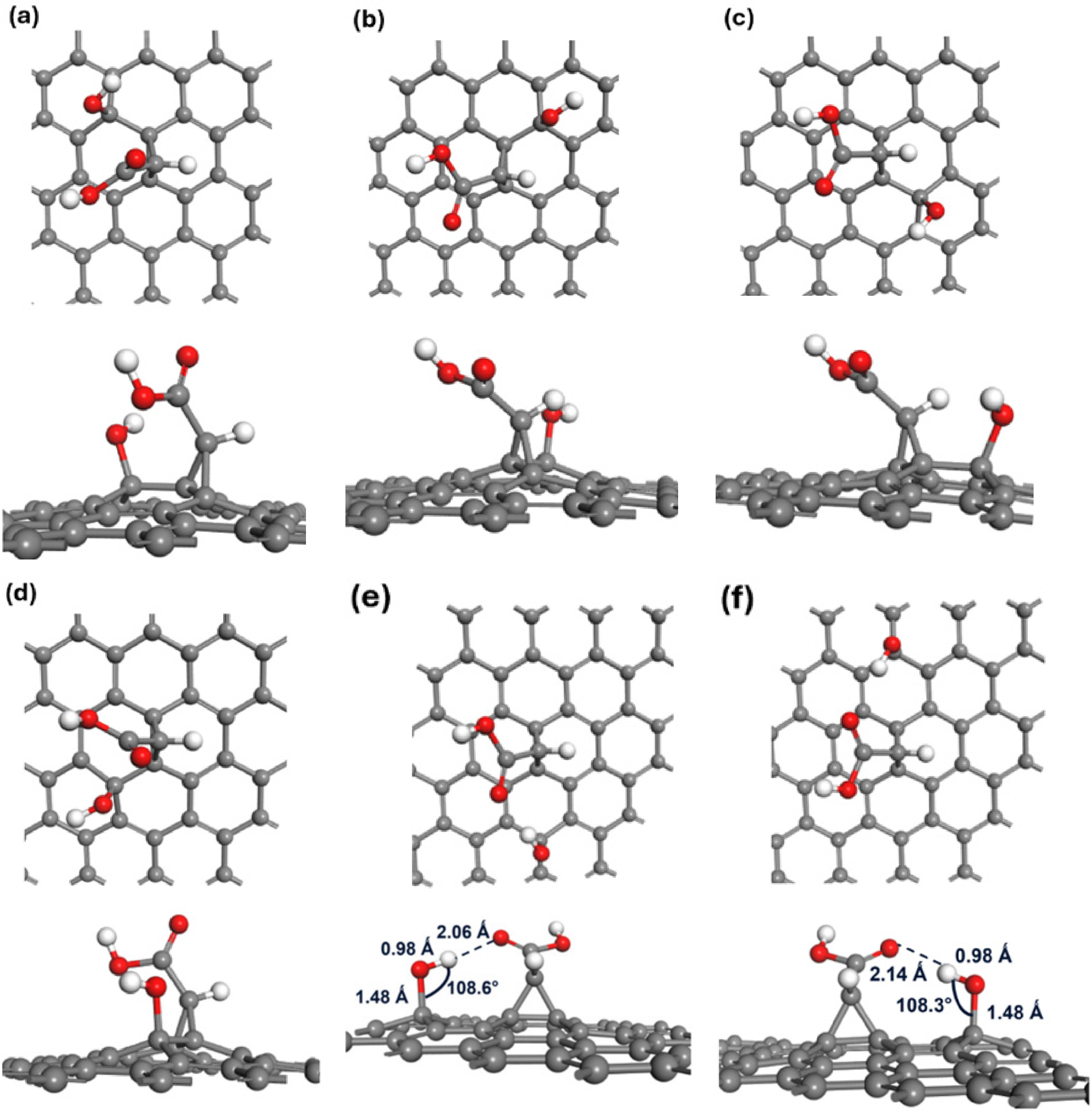

Figure S2. Top and side views for the most stable structures -OH group on above carbene graphene surface (a) C1, (b) C2, (c) C3, (d) C4, (e) C13, and (f) C14.

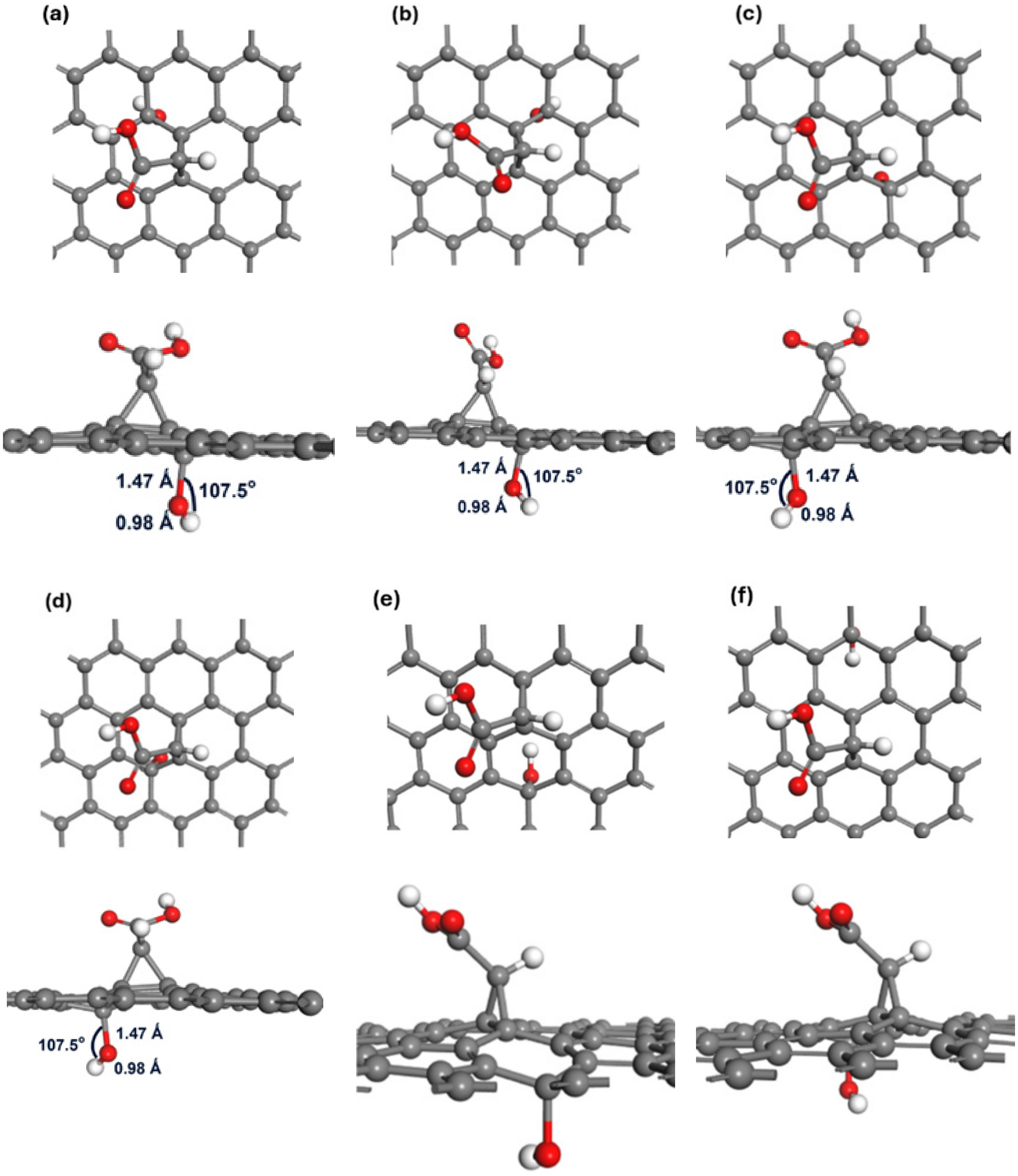

Figure S3. Top and side views for the most stable structures -OH group on down carbene graphene surface (a) C1, (b) C2, (c) C3, (d) C4, (e) C13, and (f) C14.

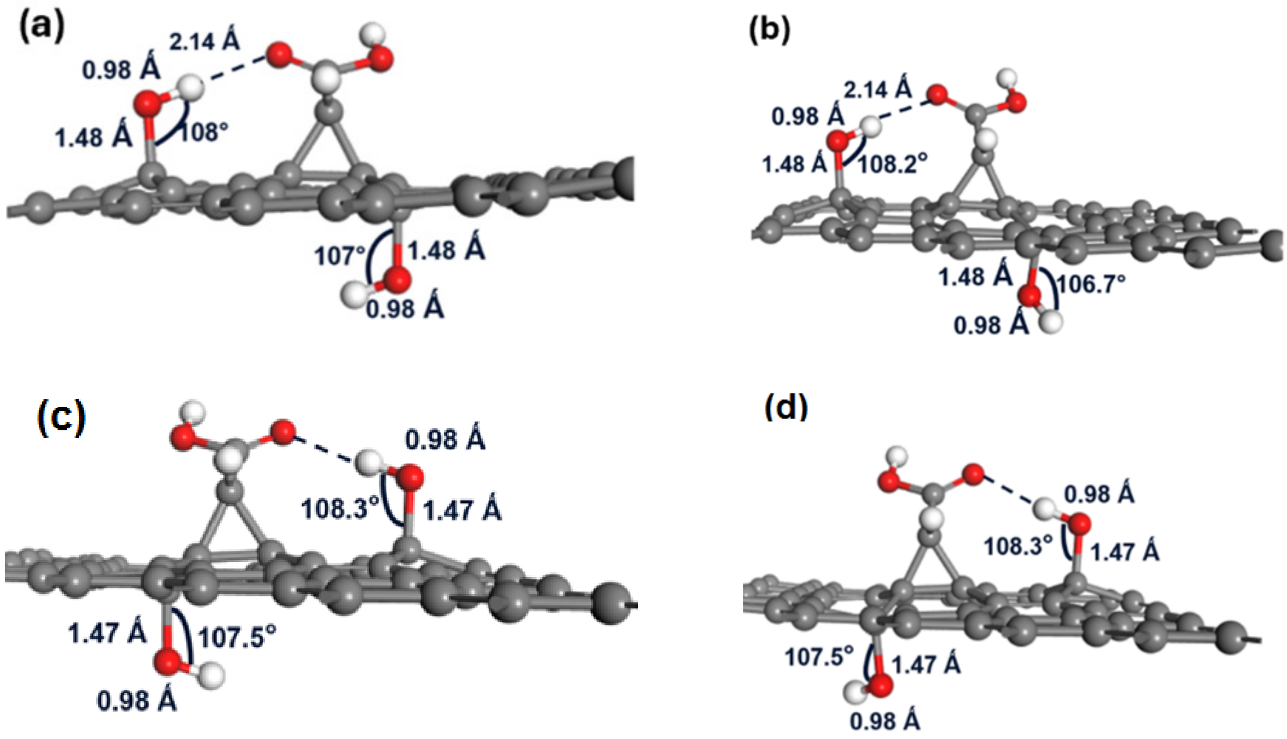

Figure S4. The highest binding energy positions for two OH group modified carbene graphene surface (a) C1 and (b) C2 with fixed position 13 above (c) C3 and (d) C4 with fixed position 14 above.

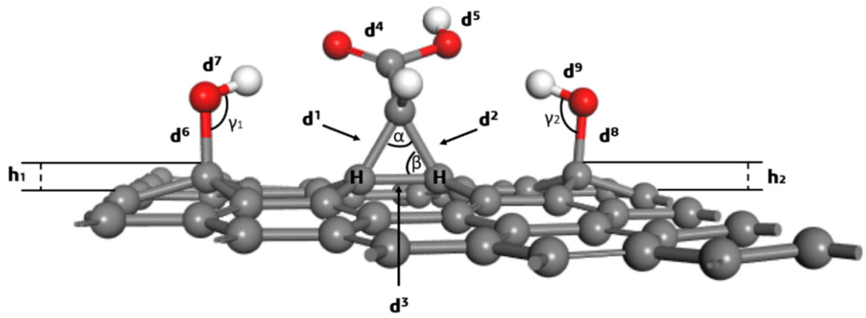

Figure S5. Symbols of the geometric parameters after modification carbene graphene by two OH group in positions 13 and 14 above the surface.

Table S1. Binding energy (E_b) for the 17 positions of two -OH group with (a) position 13 (b) position 14 modified carbene graphene surface

**Position**	**a**	**b**
	E_b(eV)	E_b(eV)
**1**	-1.60	-0.90
**2**	-1.67	-0.90
**3**	-0.99	-1.63
**4**	-0.89	-1.51
**5**	-1.27	-0.72
**6**	-1.32	-0.79
**7**	-0.85	-1.34
**8**	-0.77	-1.21
**9**	-0.74	-1.42
**10**	-0.71	-1.14
**11**	-1.20	-0.64
**12**	-1.42	-0.80
**13**	-	-1.97
**14**	-1.97	-
**15**	-0.87	-1.13
**16**	-0.70	-1.38
**17**	-0.76	-0.99

Table S2. Binding energy (E_b) for the 17 positions of two OH group above and below carbene graphene surface with (a) fixed position 13 above (b) fixed position 14 above

	**a**	**b**
	E_b(eV)	E_b(eV)
**1**	-2.09	-1.24
**2**	-2.14	-1.29
**3**	-1.37	-2.12
**4**	-1.31	-2.09
**5**	-1.87	-0.84
**6**	-1.41	-0.79
**7**	-0.81	-1.37
**8**	-0.85	-1.87
**9**	-0.84	-1.87
**10**	-0.81	-1.36
**11**	-1.39	-0.79
**12**	-1.87	-0.84
**13**	-1.84	-1.76
**14**	-1.76	-2.21
**15**	-0.81	-1.32
**16**	-0.73	-1.47
**17**	-0.86	-1.27

Table S3. Binding energy (E_b) for the 17 positions of two OH group modified carbene graphene surface with fixed position 3 below the surface

	**E_b(eV)**
**1**	-2.01
**2**	-2.50
**3**	-
**4**	-1.57
**5**	-1.78
**6**	-1.86
**7**	-0.87
**8**	-0.90
**9**	-0.84
**10**	-0.91
**11**	-1.34
**12**	-1.53
**13**	-1.00
**14**	-1.63
**15**	-0.91
**16**	-0.82
**17**	-0.86
